# Should the Contribution of One Additional Lame Cow Depend on How Many Other Cows on the Farm Are Lame?

**DOI:** 10.3390/ani7120096

**Published:** 2017-12-11

**Authors:** Peter Sandøe, Björn Forkman, Franziska Hakansson, Sine Norlander Andreasen, Rikke Nøhr, Matt Denwood, Thomas Bøker Lund

**Affiliations:** 1 Department of Veterinary and Animal Sciences, University of Copenhagen, 1870 Frederiksberg C, Denmark; bjf@sund.ku.dk (B.F.); fh@sund.ku.dk (F.H.); sinora@live.dk (S.N.A.); md@sund.ku.dk (M.D.); 2 Department of Food and Resource Economics, University of Copenhagen, 1958 Frederiksberg C, Denmark; rinohr79@gmail.com (R.N.); tblu@ifro.ku.dk (T.B.L.)

**Keywords:** animal welfare, aggregation, Welfare Quality^®^, lameness, expert perception, welfare assessment

## Abstract

**Simple Summary:**

To give a complete picture of animal welfare on a farm, different welfare measures must be combined. The aim of this paper is to assess the method by which this is achieved within the EU-funded project Welfare Quality^®^ (WQ). According to the protocols of WQ, individual animals with welfare problems contribute disproportionately more to a lower animal welfare score when they are associated with farms with an otherwise low prevalence of welfare problems compared to farms with a higher prevalence. As a consequence, the addition of a single lame cow on a farm with relatively few lame cows will have a greater consequence for the welfare score than on a farm with more lame cows. The stated aim of this aspect of the protocols is to prevent welfare problems being masked as a result of attaining better scores in other areas. By combining a case study of 44 Danish dairy farms and a questionnaire study of over 150 animal welfare experts, we test whether the system successfully prevents masking of problems that experts find to be unacceptable. Our findings indicate that this is not the case, and we conclude that better methods of summarizing farm-level animal welfare measures are required.

**Abstract:**

Welfare Quality^®^ proposes a system for aggregation according to which the total welfare score for a group of animals is a non-linear effect of the prevalence of welfare scores across the individuals within the group. Three assumptions serve to justify this: (1) experts do not follow a linear reasoning when they assess a welfare problem; (2) it serves to prevent compensation (severe welfare problems hidden by scoring well on other aspects of welfare); (3) experts agree on the weight of different welfare measures. We use two sources of data to examine these assumptions: animal welfare data from 44 Danish dairy farms with Danish Holstein Friesian cows, and data from a questionnaire study with a convenience sample of 307 experts in animal welfare, of which we received responses from over 50%. Our main results were: (1) the total group-level welfare score as assigned by experts is a non-linear function of the individual animal welfare states within the group; (2) the WQ system does not prevent what experts perceive as unacceptable compensation; (3) the level of agreement among experts appears to vary across measures. Our findings give rise to concerns about the proposed aggregation system offered by WQ.

## 1. Introduction

Welfare Quality^®^ (WQ) proposes a system for aggregating animal welfare indicators that goes against a strong dogma in animal welfare science, i.e., that welfare is defined by the state of an individual animal [1], and that the welfare of a group is therefore arguably equal to the sum of the welfare of the individual animals within that group. In WQ, aggregation is completed in three steps: from measures to criteria; from criteria to principles; and, finally, from principles to an overall score. Even at the most basic level involving individual measures of welfare, e.g., lameness in dairy cows, an aggregation is undertaken whereby the resulting total welfare score for a group of animals has a non-linear relationship with the prevalence of measured problems of the individuals within the group. Therefore, according to the WQ system, the effect of an additional lame cow on the total welfare score is not fixed, but rather depends on the number of other lame cows on the farm.

The WQ aggregation system appears to be based on three assumptions: (1) that experts view the total welfare score for a group of animals as a non-linear effect of the prevalence of welfare scores of the individuals within the group; (2) that this kind of system will prevent (or severely limit) compensation, i.e., the opportunity to hide severe welfare problems by scoring well on other aspects of welfare; (3) that experts agree on the relative weight that should be assigned to different welfare indicators during aggregation.

Details of the expert consultations that form the basis of the first assumption are not perfectly clear from the literature, but from existing sources [2] the following can be inferred: in order to transform different measures relating to a certain indicator into numbers, selected scientists from the WQ team (typically four to eight individuals) were consulted. In the case of lameness, for example, two consultative steps were taken. In the first step, the experts were consulted about the relative weight of the two conditions measured; mild and severe lameness (for more on the definition of these measures, see [3]). It was decided that mild lameness should be given a weight of 2 and severe lameness a weight of 7, so that in terms of welfare impact, one severely lame cow would be equal to three and a half mildly lame cows. On this basis, an index for lameness ranging from 0 to 100 was defined. This index score is a linear and additive function of the relative number of lame cows adjusted with the given weights. In a simple situation where cows are either walking normally (i.e., not lame at all) or are severely lame, the index will be a simple linear function of the number of non-lame cows. Using this approach, a farm with no lame cows will receive an index score of 100, and a farm where half of the cows are severely lame will receive an index score of 50. In the second step of the expert consultation, the index score was transformed into a welfare score by asking the experts to assign weights to different levels of welfare problems. Although this exercise is not fully described, the stated outcome was “that experts do not follow a linear reasoning, e.g., for a given disorder a 10% increase does not yield the same decrement in expert scores at the bottom of the 0–100 scale (where most animals get this disorder) than at the top of the scale (when most animals are normal)” ([2], pp. 23–24). 

Based on these findings, the WQ team in charge of defining aggregation developed a non-linear mathematical function to transform welfare indexes into welfare scores. The index and the welfare score are the same at both extreme ends of the scale. Therefore, for a farm where none of the cows are lame, both the index and the welfare score for lameness will be 100, and in a farm where all cows are severely lame, both will be 0. However, between these two ends of the curve, the welfare score will decrease much more quickly than the index. For example, on a farm with 90% non-lame cows and 10% severely lame cows, the index score will be 90, whereas the lameness score will be 48. If 80% of the cows are non-lame and 20% are severely lame, the index will drop to 80 and the welfare score to 29. Likewise, if 70% of the cows are non-lame and 30% are severely lame, the index will drop to 70 and the welfare score to 21. The addition of a lame cow on a farm with a low occurrence of lameness will, therefore, contribute much more to the welfare score than an additional lame cow on a farm with a high occurrence of lameness. 

Assumption (2), that the system will prevent compensation, provides one of the reasons that may underlie the experts’ reactions. Giving more importance to the first lame cow than to the last may give the farmer a strong incentive to aim for a very low prevalence of lameness. A similar consideration applies in terms of aggregating across measures. Here, the concern would be that a farmer could have severe welfare problems on the farm (e.g., in the form of a high prevalence of severe lameness), yet these could be compensated by the farm scoring well on other measures, resulting in a decent welfare score overall. 

This issue is, of course, heavily loaded with ethical considerations. A group of WQ researchers [4] notes that different schools of ethical thinking will give very different answers to the issue of compensation. For example, from a utilitarian view the morally right action is the one that maximizes total welfare, whereas other views have a different focus, e.g., on the plight of those worst off. From the utilitarian perspective, according to the interpretation of the previously mentioned WQ researchers, severe suffering in some individuals may be accepted if the group has a high level of welfare on average. An alternative view may stress the need to ensure a decent minimum level of welfare for all individuals, even if a higher total level of welfare could be achieved by a distribution where some animals suffer. Of course, the assumptions made here can be questioned: it may be argued that the suffering of a few cows weighs very heavily on a utilitarian scale whereas the resulting welfare benefits for the many cows in an intensive production system may be too trivial to weigh in terms of suffering. 

In the previously mentioned paper, the group of WQ researchers promote a pragmatic approach, according to which prevention of animal suffering is “considered to be of prime importance while accepting that a certain percentage of animals will suffer” ([4], p. 1190). The researchers indicate that the judgement of the experts consulted, “was more positive when all animals were in medium conditions than when some animals were in very poor conditions and some others in excellent conditions (e.g., an increase in animals which were not lame never outbalanced an increase of the same extent in animals which were severely lame)” ([4], p. 1190). These expert consultations therefore seem to support the use of weighting in the system in order to limit compensation, but so far only anecdotal evidence has been presented to support the assumption that experts view the acceptability of welfare problems as a non-linear effect of the welfare problems in individual animals on a farm.

An expression of good intentions is not the same as proof of concept. Thus, it is unclear whether WQ has implemented a scoring system that follows experts’ non-linear reasoning relating to an unacceptable level of a welfare problem. Therefore, it remains to be seen whether the WQ system set up will prevent the type of compensation described above. So far, we have described the first step of the aggregation procedure, where an index for the occurrence of a certain kind of welfare problem is transformed into a welfare score. The first step is followed by three further steps of aggregation: in the protocol concerning dairy cows, 29 welfare measures are aggregated into 12 criteria, these are in turn aggregated into 4 principles, and finally the principle scores are aggregated into an overall assessment score that holds four possible values. To date, one study [5] has found that, despite good intentions, welfare problems that are perceived as important by welfare experts (notably high levels of severely lame cows) are in most cases reduced in importance as a result of compensation during the WQ aggregation process. To the best of our knowledge, no study has attempted to follow compensation through the different steps of aggregation in WQ. 

Regarding assumption (3), that experts agree on the relative weight that should be assigned to different welfare indicators, it is a clear ambition of WQ to use welfare measures that have been validated. A minimum requirement here is that all measures used are perceived by experts as being valid, i.e., they possess face validity. This relates to the issue of aggregation in that agreement regarding how to add up different measures presupposes reasonable agreement regarding the face validity of measures that go into the weighting. If there are differing levels of face validity for a substantial number of measures, this suggests that there will also be differences when it comes to agreement on weighting. Knierim and Winckler [6] claim that all measures incorporated in WQ besides reliability possess face validity, but do not give evidence for the face validity. Thus, no study of expert perception is presented to support the assumption about face validity, and to our knowledge no such study has yet been undertaken. This assumption is, therefore, based only on anecdotal evidence. 

In this paper, we will present findings concerning the validity of these three assumptions. In relation to assumption (1), we will present results from a questionnaire study that investigated the weights given by a group of experts to different prevalence of lameness in dairy cows based on a scale of acceptability. This indicates the extent to which there is evidence of non-linearity in how experts perceive acceptable levels of welfare problems, using an example of lameness in dairy cows. In short: “*is perceived expert acceptability of welfare problems non-linear?*” These data also allow us to assess the extent to which, according to experts, the varying prevalence of one condition (mild lameness) can affect the welfare impact of a given prevalence of another condition (severe lameness). In short: “*is perceived expert acceptability of welfare problems non-additive between different welfare conditions?*” To address assumption (2), we will present a case study, namely of lameness in cows, and examine the extent to which the aggregated welfare scores reflect the expert opinions of an acceptable level of lameness. In short: “*will unacceptable compensation be prevented?*”. To achieve this, we combine two sources of data: 44 randomly selected Danish dairy farms that were assessed according to the Welfare Quality^®^ protocol, and the mentioned questionnaire study asking animal welfare experts about the acceptability of different prevalence of lameness in farms. To address assumption (3), we will present data revealing how different experts perceive the validity of different measures. In short: “*does the level of agreement among experts vary across measures?*”.

## 2. Materials and Methods 

### 2.1. Farm Data

Animal welfare data were collected from 44 Danish dairy farms with loose-housed Danish Holstein Friesian cows. The data were collected as part of a study comparing different welfare assessment protocols. The sampling population consisted of farms matching the following criteria: Danish Holstein Friesian breed; more than 50 cows on the farm; loose housing. The resulting 63 farms were contacted by telephone to see if they were interested in participating. If they were interested, they received a letter describing the project. All 63 farmers agreed to receive more information about the study. A week after receiving the letter, the farmers were once again contacted by telephone. At this stage, 44 farms agreed to participate, giving a response rate of 70%. There were 8106 cows in total, with a mean of 184 cows on each farm (min. 101, max. 452). Two of the farms were organic and there was a good spread of housing and milking systems: Four of the farms had deep bedding, whereas the remainder used cubicles, with 12 using rubber mats as bedding material, 17 using mattresses, eight using sand, two using latex, and one using straw. Twenty-seven of the farms milked in a parlor, and 17 used automatic milking (i.e., using a robot). 

A full welfare assessment was made on each farm using the Welfare Quality^®^ protocol for dairy cattle (Welfare Quality, 2009). The WQ protocol for dairy cattle consists of 29 measures that are carried out on-farm [7]. The 29 measures are used to calculate 12 criteria, which are aggregated into four principles, and these principles are then translated into an overall assessment. The number of cows (sample size) to be assessed was determined by the WQ protocol.

On-farm assessment was carried out by a trained WQ assessor, and started approximately 15 min after morning feeding. For the farms participating in this study, the assessment started between 04:15 and 09:00. For a full description of the Welfare Quality^®^ protocol and assessment, see [7].

One of the 29 measures in the WQ protocol assesses lameness. In order to assess the gait score of the cows, they were led in a straight line on a hard, level, non-slippery surface on which they would normally walk [7], as described in the WQ protocol. The recommended sample size could not be fully met on three of the farms, as it was not possible to get the recommended number of cows to walk on a hard, level, non-slippery surface. However, these farms were included in the analysis.

The cows were assessed from the side and from behind. All cows were scored on an individual level: non-lame cows (i.e., cows where the timing of steps and weight bearing were equal on all four feet) were given a score of 0, lame cows (i.e., cows with an imperfect temporal rhythm in stride that created a limp) were given a score of 1, and severely lame cows (i.e., cows with a strong reluctance to bear weight on one or more limbs) were given a score of 2 [7].

### 2.2. Questionnaire Data

#### 2.2.1. Target Demographic

The second dataset is derived from a questionnaire study with a convenience sample of experts in animal welfare. The participating experts comprised controllers/inspectors, veterinarians, researchers and consultants, who were recruited from six European countries through the professional network of the first and second authors. A number of key individuals were contacted and e-mail address lists of relevant experts were gathered. 

An online questionnaire was developed (available as supplementary material—see more below) and a pilot test of the questionnaire was conducted on eight animal experts during March 2015. An e-mail with a link to the online questionnaire was then distributed to a total of 307 identified animal experts, and data collection was carried out between 7th April and 6th July 2015. Two reminder e-mails were also sent out during this period.

#### 2.2.2. Expert Rating of the Acceptability of Different Levels of Lameness in Cattle

The experts were given the following description of two possible levels of lameness in dairy cattle in combination with video clips:

“*The following questions concern various conditions of lameness in herds of dairy cattle. Lameness describes an abnormality of movement and is caused by reduced ability to use one or more limbs in a normal manner. Lameness can vary in severity from reduced ability to inability to bear weight. The term ’lameness in a mild degree’ refers to a reduced ability (imperfect temporal rhythm in stride creating a limp) (see video 1). The term ’severely lame’ refers to a strong reluctance to bear weight on one limb, or to more than one limb being affected (see video 2).*”

Following this introduction, the respondents were presented with farms that had varying combinations of the prevalence of mild and severe lameness, and were asked to score these on a scale from 0 to 10, where the anchor points were 0 = “Completely unacceptable” and 10 = “Highly acceptable”. In total, there were 9 scenarios varying in mild and severe lameness prevalence. In order to limit the number of questions posed to each of the respondents, each was randomly allocated 5 of these 9 scenarios.

#### 2.2.3. Expert Rating of the Perceived Validity of Different Welfare Measures Used on Cattle

The survey also looked at the perceived validity of a number of animal welfare measures comprising criteria within two of the WQ principles (good health and appropriate behavior) and two types of empirical indicators (resource-based and animal-based). Ten different dairy cattle welfare measures were chosen. Respondents were presented with these measures, and asked to rate the extent to which it was a poor/good, “indicator of animal welfare”, using a scale of 0 to 10. The respondents were presented with two semantic anchor points: (0 = “Very poor” and 10 = “Very good”).

### 2.3. Analysis

#### 2.3.1. Questionnaire Data Cleaning

Of the 307 experts contacted, a total of 196 responded (64%). Of these, 11 respondents gave two or more nonsensical scores (defined as an increasing acceptability rating with increasing prevalence) in the section dealing with welfare acceptability, and were therefore removed from the analysis. In addition, only respondents with complete responses (containing all 5 scores for the prevalence combinations they were asked) were used for the assessment of non-linearity and non-additivity (*N* = 181, 59% response rate). For the comparison of agreement across different measures, the same 11 nonsensical respondents were removed as indicated above, and only the remaining respondents that provided a score for all 10 measures were used in the analysis (*N* = 159, 52% response rate).

#### 2.3.2. Is Perceived Expert Acceptability of Welfare Problems Non-Linear and Non-Additive?

The experts’ rating of welfare acceptability at different levels of mild and severe lameness was analyzed using a cumulative link mixed model with logit link and equidistant thresholds. The ordinal score (between 0 and 10) was used as the response, with a linear fixed effect of the prevalence of mild lameness, a separate linear fixed effect of the prevalence of severe lameness, and a random effect of respondent ID. This approach allows the median score to differ among respondents, although the model effectively assumes that the relative effect on the ordinal score of changing mild and severe prevalence does not differ among respondents. In addition to this simple model, a further three models were fit, incorporating: an interaction between the linear effects of mild and severe prevalence (non-additive model), an additional quadratic term for each of the mild and severe prevalence effects (non-linear model), and both the interaction between the linear effects of mild and severe prevalence, combined with a quadratic term for both mild and severe prevalence (non-linear and non-additive model). The best-fitting model was chosen based on Akaike’s Information Criterion (AIC; [8]). The best-fitting model was then used to generate the predicted probabilities of obtaining each given ordinal score level for a range of different mild and severe prevalence combinations. Statistical models were implemented using the ordinal package [9] for R [10], and graphs were produced using the ggplot2 package [11].

#### 2.3.3. Will Unacceptable Compensation Be Prevented?

In order to compare expert opinion on the acceptability of different lameness prevalence with the WQ scores for real farms with similar lameness prevalence, we first profiled the 44 farms according to their prevalence of mild and severe lameness. More specifically, we divided the farms into nine groups resembling the lameness prevalence that the experts were prompted about in the questionnaire. For each combination of mild and severe prevalence, the ordinal mixed effects model detailed above was used to generate predicted probabilities that an “average” respondent would rate the scenario with an acceptability score in the following four categories: clearly unacceptable (scores 0–2), unacceptable (scores 3–4), acceptable (scores 5–7), and clearly acceptable (scores 8–10). For each farm profile, we then characterized the score at subsequent aggregation steps (i.e., at the criteria, principle, and overall assessment levels) and compared these to the predicted scores. 

#### 2.3.4. Does the Level of Agreement among Experts Differ Across Measures?

The variance associated with respondent was quantified for each measure using a cumulative link random effects model similar to that detailed above. There were no fixed effects used for this model, and respondent was used as the sole random effect. Each of the 10 measures was modelled separately. The over-dispersion in variation among the observed ordinal scores is reflected in the variance component associated with the random effect of respondent, which we interpret as the extent to which respondents disagree by more than could be expected by chance.

## 3. Results

### 3.1. Is Perceived Expert Acceptability of Welfare Problems Non-Linear and Non-Additive?

**Figure 1:**
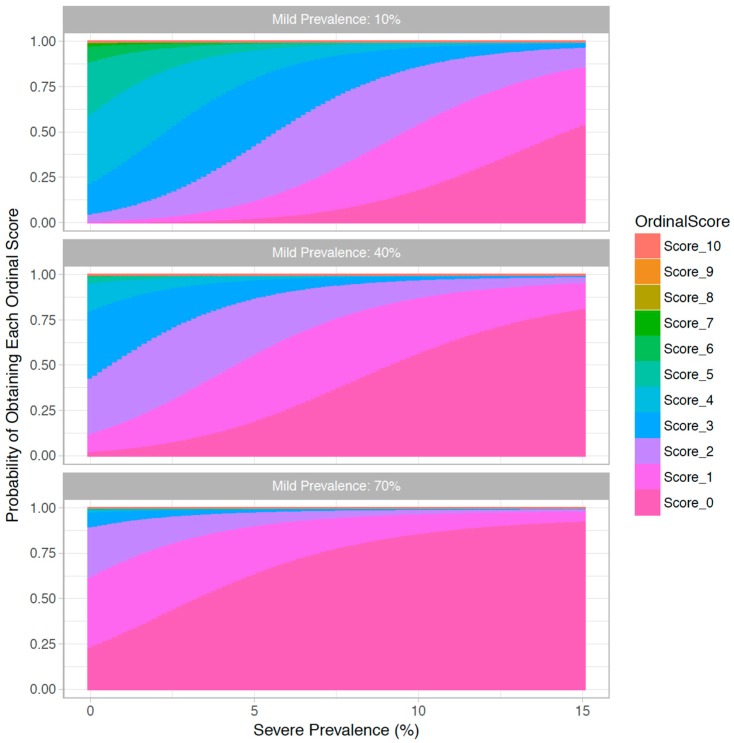
Cumulative bar charts showing the probability of obtaining each ordinal score from an “average” expert (*y* axis), given an increasing severe prevalence (*x* axis) and different levels of mild prevalence (sub-plots). Probabilities are based on a cumulative link mixed model.

**Figure 2:**
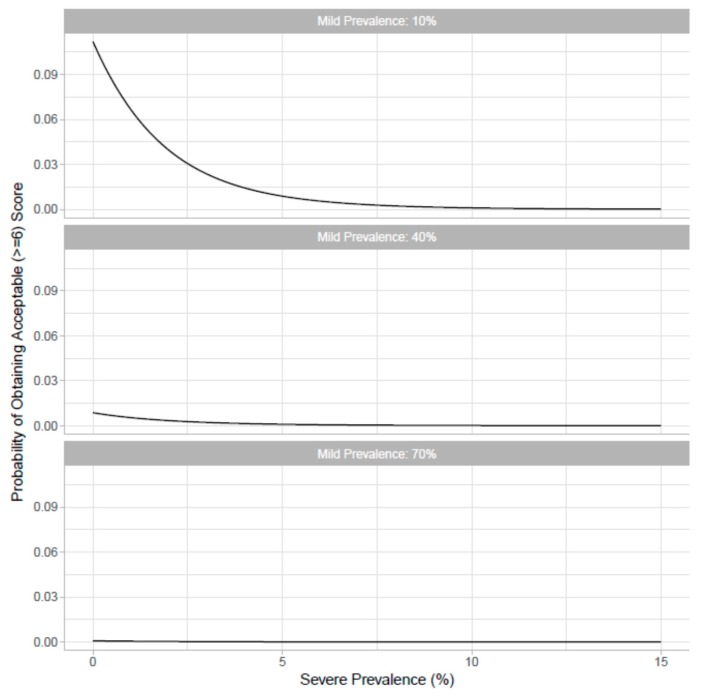
The probability of obtaining an “acceptable” score (*y* axis; defined as a score ≥6), given increasing severe lameness (*x* axis) and different prevalence of mild lameness (sub-plots). Probabilities are based on a cumulative link mixed model.

Model fit from the four models indicated that the non-additive and non-linear model was a significantly better fit to the data than the simpler models considered (Table 1). Based on the relative difference in AIC scores, the interaction between the linear effects of mild and severe prevalence appears to be more important than the quadratic effects of prevalence. Therefore, non-additivity between different welfare conditions appears to be more strongly supported by the experts than non-linearity within the prevalence of a single welfare condition. The relationship between the predicted acceptability scores (for an “average” expert) and the varying prevalence of mild and severe lameness are displayed in Figure 1. Unsurprisingly, lower scores on the ordinal scale, indicating lower acceptability, occur more frequently at a higher prevalence of both mild and severe levels of lameness. The effect of non-linearity is not easy to discern from Figure 1, so to study this we identified a threshold acceptability level (≥6) and plotted the probability of obtaining an “acceptable” score given the varying prevalence of mild and severe lameness (Figure 2). With a mild prevalence of 10% (top graph), the non-linear functional form manifests as an initially steep decrease in acceptability as the prevalence of severe lameness increases. However, this pattern only occurs as long as the prevalence of mild lameness is low. If the prevalence of mild lameness is relatively high (i.e., in the middle and lower graph of Figure 1), this non-linearity is harder to see because of the extremely low probability that the scenario would be scored as acceptable for any given severe lameness prevalence. 

### 3.2. Will Unacceptable Compensation Be Prevented?

Table 2 shows how different combinations of lameness prevalence affect the aggregated scores through the three steps of aggregation in WQ for the 44 dairy farms included in our case study. These aggregation steps are: from the level of single measures to the level of a criterion, from the level of criterion to the level of a principle, and finally from the level of principle to an overall assessment of the farm. At each level, there are four possible scores ranging from best to worst (termed “Excellent”, “Enhanced”, “Acceptable” and “Not classified”).

The criterion absence of injuries (Criterion 6) includes the measures integument alterations and the assessed level of lameness. As seen from the “totals”, the majority of farms are in the two worst categories, and no farms are in the best category, meaning that no compensation has taken place. At the next level of aggregation (principle level), absence of injuries is combined with two other criteria (absence of disease and absence of pain induced by management procedures) into the principle good health. All farms perform well in terms of the latter criterion, thanks to compliance with Danish animal welfare legislation, and most farms also fare reasonably well in terms of disease prevalence. As a consequence, no farms end up in the lowest category (not classified). Finally, when aggregating to the overall score, all four principles (good health, good feeding, good housing and appropriate behavior) are aggregated. Here, one farm is not classified, while the remainder are evenly divided across the intermediate enhanced and acceptable categories.

It is also instructive to examine whether the degree of compensation found here would be considered acceptable based on the expert questionnaire. Table 3 shows the predicted probability that an average expert would classify different levels of lameness within the acceptability categories indicated.

Based on this, the probability that an average expert would classify the prevalence of lameness indicated in the questionnaire to be clearly acceptable (8–10) is extremely small. It is only for the lowest prevalence of mild lameness in combination with no severe lameness (i.e., profile 1) for which there is an appreciable probability that an average expert would give a classification of partly acceptable. There is a high probability that combinations of lameness reflected by profiles 3, 4, 6, 7, 8 and 9 would be classified as clearly unacceptable. The remaining profiles 1, 2 and 5 comprise only 22.7% (95% CI: 12.9–37.1%) of the farms. In addition, there is an extremely high probability (between 97.1% and 99.7%) that an average expert would find profiles 6, 7, 8 and 9, comprising 36.4% (95% CI: 23.8–51.2%) of the farms, to be clearly unacceptable. In contrast, less than 3% of farms are in the unacceptable (“not classified”) category in the overall WQ assessment, so it can be concluded that the degree of compensation found in our case study would not generally be considered acceptable by the experts.

### 3.3. Does the Level of Agreement among Experts Differ across Measures?

**Figure 3:**
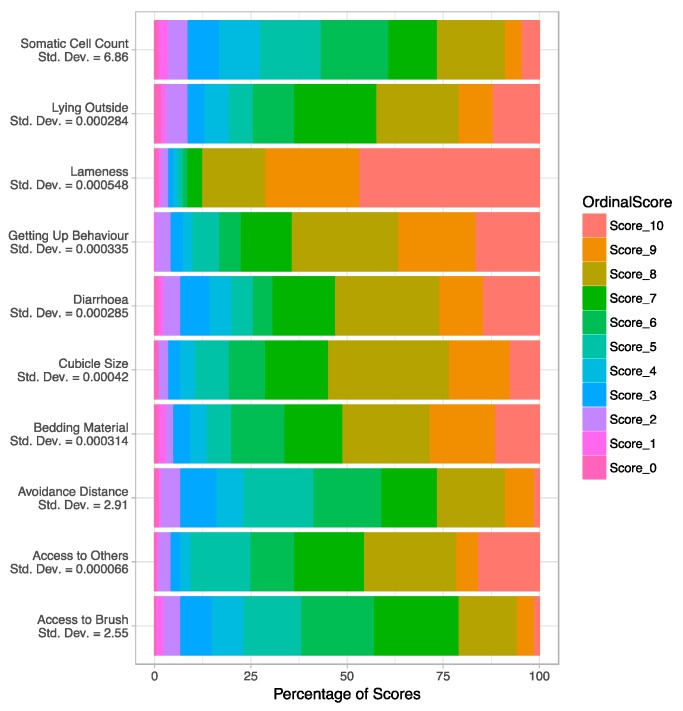
Cumulative horizontal bar charts showing the proportion of scores returned in the raw data (*x* axis) for each measure (*y* axis). Estimates of the standard deviation associated with the random effect of respondent obtained from the cumulative link random effects model are shown underneath the measure name on the *x*-axis labels.

Figure 3 shows the proportion of scores returned for each measure (raw data), including estimates of the standard deviation associated with the random effect of respondent (based on the model). The distribution of scores is qualitatively very similar for all measures except somatic cell count (SCC), access to brush, and avoidance distance, which seem to have a lower median and more substantial variation (i.e., disagreement among experts) than the other measures. Lameness differs from the other measures in that the median score is higher, but the variation across scores does not seem to differ from that of the other measures. The random effect estimate is higher for SCC (standard deviation = 6.86) than for the other measures, indicating that the highest degree of disagreement among experts is likely to be for SCC. The corresponding estimate is relatively similar between the measures of avoidance distance (standard deviation = 2.91) and access to brush (standard deviation = 2.55). For all other measures, the random effects estimate is close to zero, indicating that all of the observed variation among experts can be explained by random variation in scores (i.e., there is no evidence for systematic variation among experts within this data sample). However, it is not possible to generate confidence intervals for these random effect estimates, so extreme caution must be used when extrapolating these results from the observed sample to the population level.

## 4. Discussion

Our findings show that: (1) there is significant evidence that the average expert will evaluate the total welfare score for a group of animals as a non-linear function of the prevalence of welfare scores across individuals within the group, and also that the combination of different welfare conditions is non-additive; (2) our case study demonstrates a situation in which the WQ system does not prevent compensation at a level that our experts would perceive as unacceptable, i.e., unacceptable welfare problems were effectively hidden by good scores for other aspects of welfare; (3) there is some evidence that the level of agreement among experts varies across measures. 

Findings related to (1), the non-linear function, replicate what has been claimed by researchers from WQ. However, to the best of our knowledge we are the first to provide evidence for this assumption. Our findings in relation to (2), compensation, are novel in that no study has as yet combined the outcomes of measures on the farm with data on experts’ perceived acceptability. However, our findings are also very much in line with those of other researchers. For example, in a study of 196 dairy herds, [5] found that “except for one herd, a high prevalence of (severely) lame cows did not result in herds being classified as unacceptable” (p. 6271). They combined this with a reference to two other studies [12,13], showing that animal welfare experts rank “lameness as the most important measure of dairy cattle welfare” (p. 6271). They also show the mechanisms in the WQ system that give rise to this problematic sort of compensation. In addition, our findings are in line with [14], who found a low level of correspondence between the overall WQ welfare score and scoring by experts. Our findings relating to (3), agreement between experts, point in the same direction. However, more work must be done to establish whether these differences in agreement are biologically significant/meaningful; for example, whether the observed variation among experts would result in a quantitatively different outcome (in terms of WQ or other similar protocols) for a given scenario. 

In essence, points (2), compensation, and (3), agreement between experts, give rise to concerns about the proposed aggregation system offered by WQ, whereas (1) seems to speak in favor of it. However, there are independent reasons to be skeptical about how experts are used in defense of (1) for two reasons: firstly, experts were asked to score the aggregated welfare on a scale that included the terms “acceptable” and “unacceptable” as its anchor points. Therefore, a welfare score (“how bad for the cows?”) is transformed into an ethics score (“how acceptable?”). However, the respondents were experts in the field of welfare, not of ethics, and the question of whether any individual can be an expert in ethics in any relevant sense is open to debate. It is important here to point to the results of a study [15] that found that three different groups of stakeholders, “farmers, citizens and vegetarians”, disagreed with both the WQ aggregation procedure and each other. 

## 5. Conclusions

In conclusion, our results indicate that the methods for aggregation used in WQ are quite problematic, and specifically fail in their stated aim of avoiding compensation between measures. We also suggest that the link between ethical judgements and the weighting process become completely opaque as a result of the weighting being done in three steps. Even though experts may know what they are doing when they indicate their moral acceptance of the levels of welfare problems, it is very unlikely that they will be able to take the whole picture into account when aggregating in three more iterations across numbers that reflect weightings done at lower levels.

## Figures and Tables

**Table 1:** Comparison of model fit on Akaike’s Information Criterion (AIC) for four models describing the relationship between lameness prevalence and the acceptability of the scenario according to expert opinion. Smaller AIC scores indicate a better-fitting model.

Model	AIC
Simple model (linear effects of mild and severe prevalence with no interaction)	2404.6
Non-additive model (linear effects of mild and severe prevalence with an interaction)	2383
Non-linear model (linear and quadratic effects of mild and severe prevalence with no interaction)	2402.8
Non-additive and non-linear model (linear and quadratic effects of mild and severe prevalence with an interaction between the linear effects)	2375.8

**Table 2:** The 44 dairy cattle farms profiled according to lameness (in prevalence of mild and severe lameness), and share of the profiled farms that are assigned as excellent, enhanced, acceptable, and not classified at the criteria, principle and overall assessment.

Lameness Profile			Level 1: Criteria 6 (Absence of Injuries) (Row %)				Level 2: Principle 3 (Good Health) (Row %)				Level 3: Overall (Row %)			
	%	(*n*)	Excellent	Enhanced	Acceptable	Not Classified	Excellent	Enhanced	Acceptable	Not Classified	Excellent	Enhanced	Acceptable	Not Classified
Profile 1: Mild lameness: 5–15%/severe lameness: 0%	4.5	(2)	0	100	0	0	0	100.0	0	0	0	50	50	0
Profile 2: Mild lameness: 15–40%/severe lameness: 0%	13.6	(6)	0	50.0	50.0	0	0	50.0	50.0	0	0	66.7	33.3	0
Profile 3: Mild lameness: 40–70%/severe lameness: 0%	31.8	(14)	0	7.1	92.9	0	0	0	100.0	0	0	42.9	57.1	0
Profile 4: Mild lameness: >70%/severe lameness: 0%	9.1	(4)	0	0	50.0	50.0	0	0	100.0	0	0	25	75	0
Profile 5: Mild lameness: 15–40%/severe lameness: 5–15%	4.5	(2)	0	0	100.0	0	0	0	100.0	0	0	50	50	0
Profile 6: Mild lameness: 40–70%/severe lameness: 5–15%	15.9	(7)	0	14.3	85.7	0	0	14.3	85.7	0	0	71.4	28.6	0
Profile 7: Mild lameness: >70%/severe lameness: 5–15%	4.5	(2)	0	0	50.0	0	0	0	100.0	0	0	50	0	50
Profile 8: Mild lameness: 15–40%/severe lameness: >15%	9.1	(4)	0	0	75.0	25.0	0	0	100.0	0	0	75	25	0
Profile 9: Mild lameness: 40–70%/severe lameness: >15%	6.8	(3)	0	0	66.7	33.3	0	0	100.0	0	0	0	100	0
Total	100	(44)	0	15.9	72.7	11.4	0	13.6	86.4	0	0	50	47.7	2.3

**Table 3:** Expert opinion about the acceptability (in percent) of prevalence of lameness in dairy cattle ^A^.

Prevalence of Lameness	Clearly Unacceptable (Score 0–2)	Partly Unacceptable (Score 3–4)	Partly Acceptable (Score 5–7)	Clearly Acceptable (Score 8–10)
Mild lameness: 10%/severe lameness: 0%	5.1	55.2	39.2	0.4
Mild lameness: 40%/severe lameness: 0%	43.5	52.6	4.4	0
Mild lameness: 70%/severe lameness: 0%	90	2.7	0.1	0
Mild lameness: 10%/severe lameness: 5%	42.9	52.1	4.3	0
Mild lameness: 40%/severe lameness: 5%	87.3	12.2	0.5	0
Mild lameness: 70%/severe lameness: 5%	98.1	0.7	0	0
Mild lameness: 10%/severe lameness: 15%	97.1	9.5	0.4	0
Mild lameness: 40%/severe lameness: 15%	99.2	1.8	0.1	0
Mild lameness: 70%/severe lameness: 15%	99.7	0.2	0	0

^A^ The reported percentages are calculated predicted probabilities for an average expert based on the mixed effects model.

## Supplementary Materials

The following are available online at www.mdpi.com/2076-2615/7/12/96/s1: questionnaire from the sociological study, two datasets from the sociological study, and one dataset from the animal study.
