# Supplementary material for: Should the Contribution of One Additional Lame Cow Depend on How Many Other Cows on the Farm Are Lame?
Source: Animals (Basel). 2017 Dec 11;7(12):96. doi: 10.3390/ani7120096 (PMC5742790; doi:10.3390/ani7120096)
Supplement: Supplementary file 1 [file animals-07-00096-s001.zip › Questionnaire Final - Animal Welfare Experts.pdf]

Thank you for participating in this survey which is conducted as part of a Danish project by the University of Copenhagen University, University of Aarhus, and The Danish Veterinary & Food Administration. The focus of this part of the project is to examine how experts view animal welfare concerning cattle and pigs at the farm level. In this project the term “welfare" means the balance between positive and negative experiences in the animals. The animals have a high level of welfare if they, relatively speaking, have few negative experiences, for example of pain, fear, frustration and discomfort, and many positive experiences, for example of pleasure, play and relaxation.

Thus the focus here is on the welfare of the animals, not on compliance with animal welfare legislation. So we ask you not to consider whether what you are asked about is legal or not, but rather to focus on how you think the welfare of the animals in question is affected.

Furthermore, although some of the following conditions in animal herds might come across as a bit unrealistic, please keep in mind that they solely represent examples in order to elucidate how experts assess animal welfare.

Thanks!

----------------- NY SIDE--------------------------

1. What is your age? (Userneed: single response)

24 years old or under: __ 25 - 34 years old: __ 35 - 44 years old: __

45 - 54 years old: __ 55 - 64 years old: __ 65 - 74 years old: __

75 years or older: __

-----------NY SIDE-------------

2. What is your gender? (Userneed: single response)

Male: ___ Female: ___

-----------NY SIDE-------------

3. In which country are you residing? (Userneed: single response)

Denmark: ___ Sweden: ___ Finland: ___

England: ___ Scotland: ___ Holland: ___

Other country: ___ please specify: ___

-----------NY SIDE-------------

FILTERSPØRGSMÅL 1

Do you have any professional knowledge and/or experience of either pig and/or cattle production?

Yes:___ No:___ (GO TO '99')

4. On a scale from 0-10, how would you evaluate your own professional knowledge and/or experience of cattle production?

0 1 2 3 4 5 6 7 8 9 10

No knowledge at all Highly specialized expert

5. On a scale from 0-10, how would you evaluate your own professional knowledge and/or experience of pig production?

0 1 2 3 4 5 6 7 8 9 10

No knowledge at all Highly specialized expert

-----------NY SIDE-------------

6. Are you currently working?

Yes: __ (GÅ TIL 7) No: __ (GÅ TIL FILTERSPØRGSMÅL 2)

-----------NY SIDE-------------

FILTERSPØRGSMÅL 2

I'm studying: __ Please specify the education: ________ (GÅ TIL 9.B) I'm unemployed: ___ (GÅ TIL 9.B.)

-----------NY SIDE-------------

7. Which of the following professions characterizes your *current* employment, and how many years have you worked with this? (Userneed: multiple responses)

(Tick off one or several of the options that are relevant to you)

Veterinary animal production practitioner involved with pigs and/or cattle in livestock as part of my work:___

Less than 1 year: __ 1-3 year(s): __ 4-6 years: __ 7-10 years: __ 11 years or more: __ (GÅ TIL 8)

Consultant involved with pigs and/or cattle in livestock as part of my work:___

Less than 1 year: __ 1-3 year(s): __ 4-6 years: __ 7-10 years: __ 11 years or more: __(GÅ TIL 8)

Researcher involved with pigs and/or cattle in livestock as part of my work:___

Less than 1 year: __ 1-3 year(s): __ 4-6 years: __ 7-10 years: __ 11 years or more: __(GÅ TIL 8)

Animal Welfare Controller/Inspector involved with pigs and/or cattle in livestock as part of my work:___

Less than 1 year: __ 1-3 year(s): __ 4-6 years: __ 7-10 years: __ 11 years or more: __(GÅ TIL 8)

None of the professions above:___ **Please specify your profession:**___ (GÅ TIL: 9.B.)

-----------NY SIDE-------------

8. Which kind of sector do you work in? (Userneed: single response)

Public sector: ___ Private sector: ___ Non-Profit:___ A combination: ___

Self-employed:___ Other: ___ Please specify: ___

-----------NY SIDE-------------

9. If you have had any *former* employment, which of the following professions characterizes this, and how many years did your work with this? (Tick off one or several of the options that are relevant to you) (Userneed: multiple responses) (Tick off one or several of the options that are relevant to you)

Veterinary animal production practitioner involved with pigs and/or cattle in livestock as part of my work:___

Less than 1 year: __ 1-3 year(s): __ 4-6 years: __ 7-10 years: __ 11 years or more: __ (GÅ TIL 10)

Consultant involved with pigs and/or cattle in livestock as part of my work:___

Less than 1 year: __ 1-3 year(s): __ 4-6 years: __ 7-10 years: __ 11 years or more: __(GÅ TIL 10)

Researcher involved with pigs and/or cattle in livestock as part of my work:___

Less than 1 year: __ 1-3 year(s): __ 4-6 years: __ 7-10 years: __ 11 years or more: __ (GÅ TIL 10)

Animal Welfare Controller/Inspector involved with pigs and/or cattle in livestock as part of my work:___

Less than 1 year: __ 1-3 year(s): __ 4-6 years: __ 7-10 years: __ 11 years or more: __ (GÅ TIL 10)

None of the professions above:___ (GÅ TIL 10)

9.B. Which of the following professions characterizes your *former* employment, and how many years did you work with this? (Userneed: multiple responses)
(Tick off one or several of the options that are relevant to you)

Veterinary animal production practitioner involved with pigs and/or cattle in livestock as part of my work:___

Less than 1 year: __ 1-3 year(s): __ 4-6 years: __ 7-10 years: __ 11 years or more: __ (GÅ TIL 10)

Consultant involved with pigs and/or cattle in livestock as part of my work:___

Less than 1 year: __ 1-3 year(s): __ 4-6 years: __ 7-10 years: __ 11 years or more: __ (GÅ TIL 10)

Researcher involved with pigs and/or cattle in livestock as part of my work:___

Less than 1 year: __ 1-3 year(s): __ 4-6 years: __ 7-10 years: __ 11 years or more: __ (GÅ TIL 10)

Animal Welfare Controller/Inspector involved with pigs and/or cattle in livestock as part of my work:___

Less than 1 year: __ 1-3 year(s): __ 4-6 years: __ 7-10 years: __ 11 years or more: __(GÅ TIL 10)

None of the professions above:___ (GÅ TIL 10)

-----------NY SIDE-------------

10. Have you completed levels of education beyond compulsory school?

Yes:___ (GÅ TIL SPG 11) No:___(GÅ TIL SPG 15)

-----------NY SIDE-------------

11. Have you completed a technical education?

Yes:___ (GÅ TIL SPG 12) No:___(GÅ TIL SPG 12)

-----------NY SIDE-------------

12. Have you completed a university degree?

Yes:___ (GÅ TIL SPG 13) No:___(GÅ TIL SPG 14)

-----------NY SIDE-------------

13. Have you completed a PhD degree?

Yes:___ No:___

-----------NY SIDE-------------

14. Which of the following types of higher education have you completed? (Userneed: multiple responses)

(Tick off one or several of the options that are relevant to you)

Veterinary: ___ Animal Welfare: ___ Biology: ___

Agronomy and Agriculture: ___ Other: ___ Please specify: _____________

-----------NY SIDE-------------

The following questions concern how well various animal welfare measures are able to capture the welfare at herd level for dairy cattle and calves, sows and gilts, and finishing pigs and weaners.

------------------ NY SIDE---------------------------

Dairy Cattle and the animal welfare measure 'lying outside the lying area'

The lying position of each animal is being assessed, to check whether they are lying correctly in the lying area. An animal having a hindquarter on the edge of the lying area (cubicle or deep bedded area) or lying with hindquarter (both hind legs) outside the lying area (cubicle or deep bedded area) are registered as a welfare problem. The pictures below illustrate two examples of cattle lying outside the lying area.

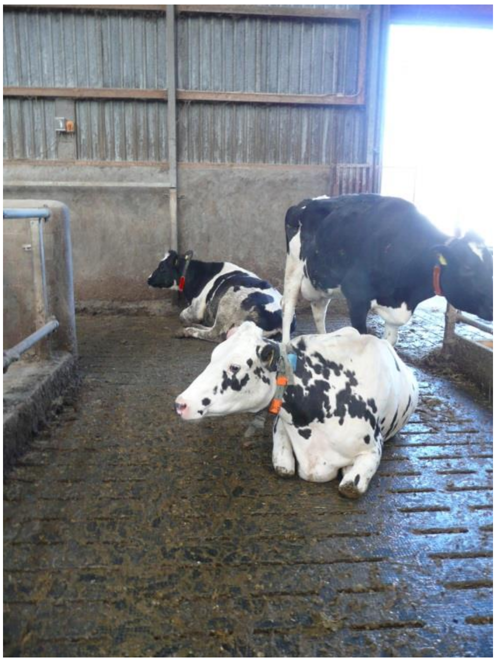

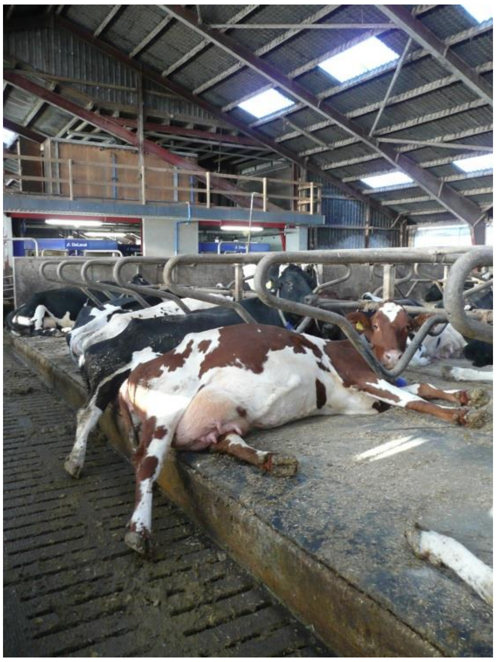

15. How would you rate *lying outside the lying area* as a measure of the underlying state of animal welfare in a dairy cattle herd?

(Please rate on a scale of 0 – 10, with 0 indicating a very poor animal welfare measure)

0 1 2 3 4 5 6 7 8 9 10

Very poor Very good

☐ Don't know

------------------ NY SIDE---------------------------

Dairy Cattle and the animal welfare measure 'getting up behaviour'

To assess the getting up behaviour of cattle, it is checked whether the animals take on a normal and smooth sequence when getting up. If the animal has a normal sequence, but with a break, stands on its front knees or has abnormal sequences (e.g. sitting like a dog), this is viewed as a sign of reduced welfare.

16. How would you rate *getting up behaviour* as a measure of the underlying state of animal welfare in a dairy cattle herd?

(Please rate on a scale of 0 – 10, with 0 indicating a very poor animal welfare measure)

0	1	2	3	4	5	6	7	8	9	10
Very poor									Very good	

☐ Don't know

----------------- NY SIDE--------------------------

Dairy Cattle and the animal welfare measure 'bedding material'

When measuring bedding material in a herd, the type and quality of the bedding is checked. A hard bedding material can cause swollen and damaged hocks and knees. Examples of soft bedding material are mattresses, straw bedding, soft rubber mats, sand and sawdust.

17. How would you rate *bedding material* as a measure of the underlying state of animal welfare in a dairy cattle herd?

(Please rate on a scale of 0 – 10, with 0 indicating a very poor animal welfare measure)

0 1 2 3 4 5 6 7 8 9 10

Very poor Very good

☐ Don't know

------------------ NY SIDE--------------------------

Dairy Cattle and the animal welfare measure 'cubicles'

It is assessed whether there is enough space in the cubicles for the cows to lie down, without being hindered by cows from adjacent cubicles. At least one cubicle per cow is required when checking this measure.

18. How would you rate *cubicles* as a measure of the underlying state of animal welfare in a dairy cattle herd?

(Please rate on a scale of 0 – 10, with 0 indicating a very poor animal welfare measure)

0 1 2 3 4 5 6 7 8 9 10

Very poor Very good

☐ Don't know

------------------ NY SIDE---------------------------

Dairy Cattle and the animal welfare measure 'milk somatic cell count'

The data of milk somatic cell count is obtained from milk records, collected from a period of three months prior to the farm visit. This measure is considered to indicate subclinical inflammation in the animal. A problem is registered when the somatic cell count is greater than 400,000.

19. How would you rate *milk somatic cell count* as a measure of the underlying state of animal welfare in a dairy cattle herd?

(Please rate on a scale of 0 – 10, with 0 indicating a very poor animal welfare measure)

0 1 2 3 4 5 6 7 8 9 10

Very poor Very good

☐ Don't know

------------------ NY SIDE---------------------------

Dairy Cattle and the animal welfare measure 'access to cow brush'

A cow brush is a rotating brush, which cows and heifers can rub themselves against. The brush performs the same task as when the cow is trying to slouch itself with its tail, but the brush is far more efficient. This measure checks whether or not there is access to at least one rotating cow brush per 50 animals.

20. How would you rate *access to cow brush* as a measure of the underlying state of animal welfare in a dairy cattle herd?

(Please rate on a scale of 0 – 10, with 0 indicating a very poor animal welfare measure)

0 1 2 3 4 5 6 7 8 9 10

Very poor Very good

☐ Don't know

----------------- NY SIDE--------------------------

Dairy Cattle and the animal welfare measure 'avoidance distance at the feeding table'

It is checked whether the animal withdraws and hence shows signs of an avoidance distance at the feeding table. Withdrawal is registered when the animal moves back, turns its head to the side, or pulls its head back trying to get out of the feeding rack. More specifically the avoidance distance is estimated as the distance between the hand and the muzzle at the moment of withdrawel.

21. How would you rate *avoidance distance at the feeding table* as a measure of the underlying state of animal welfare in a dairy cattle herd?

(Please rate on a scale of 0 – 10, with 0 indicating a very poor animal welfare measure)

0 1 2 3 4 5 6 7 8 9 10

Very poor Very good

☐ Don't know

------------------ NY SIDE---------------------------

Dairy Cattle and the animal welfare measure 'lameness'

Lameness refers to an abnormality of movement and is caused by reduced ability to use one or more limbs in a normal manner. The condition can vary in severity. An imperfect temporal rhytm in stride creating a limp is recorded as being lame in a milder degree. Animals having a strong reluctance to bear weight on one limb, or more than one limp is affected is recorded as being severely lame (see video below).

Score 2_lameness.wmv

22. How would you rate *lameness* as a measure of the underlying state of animal welfare in a dairy cattle herd?

(Please rate on a scale of 0 – 10, with 0 indicating a very poor animal welfare measure)

0 1 2 3 4 5 6 7 8 9 10

Very poor Very good

☐ Don't know

------------------ NY SIDE---------------------------

Dairy Calves and the animal welfare measure 'diarrhea'

Diarrhea is defined as loose watery manure below the tail head on both sides of the tail. When checking for diarrhea, feacal smear because of diarrhea must be present at hind legs, before registering this condition. If the animal is clean or only has feacal smear on the rear, diarrhea will not be registered.

23. How would you rate *diarrhea* as a measure of the underlying state of animal welfare in a flock of dairy calves?

(Please rate on a scale of 0 – 10, with 0 indicating a very poor animal welfare measure)

0 1 2 3 4 5 6 7 8 9 10

Very poor Very good

☐ Don't know

----------------- NY SIDE--------------------------

Dairy Calves and the animal welfare measure 'access to other calves'

It is assessed to what extent the calves have access to other calves, which fulfills their social behavioral need for contact. If a calf has no or only partial access to another calf (e.g. through a fence, see photo) it is viewed as a sign of reduced welfare.

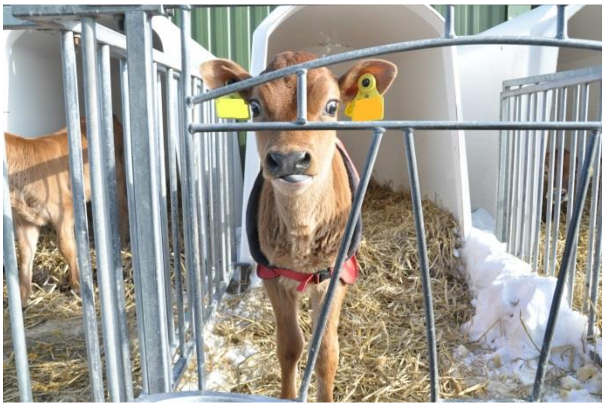

24. How would you rate *access to other calves* as a measure of the underlying state of animal welfare in a flock of dairy calves?

(Please rate on a scale of 0 – 10, with 0 indicating a very poor animal welfare measure)

0 1 2 3 4 5 6 7 8 9 10

Very poor Very good

☐ Don't know

------------------ NY SIDE---------------------------

Sows and Gilts and the animal welfare measure 'panting'

Panting in sows and gilts can be a visible sign of thermal stress. A respiratory rate of more than 28 breaths per minute in sows is considered as panting.

25. How would you rate *panting* as a measure of the underlying state of animal welfare in an sow herd?

(Please rate on a scale of 0 – 10, with 0 indicating a very poor animal welfare measure)

0 1 2 3 4 5 6 7 8 9 10

Very poor Very good

☐ Don't know

------------------ NY SIDE---------------------------

Sows and Gilts and the animal welfare measure 'cooling'

Pigs have a very limited ability to sweat and are acutely susceptible to heat stress. Cooling sprinklers and/or cooling mats can be used to ensure that pigs in buildings do not become overheated in hot weather. Only sprinklers that are working are counted as being sprinklers.

26. How would you rate *cooling* as a measure of the underlying state of animal welfare in a sow herd?

(Please rate on a scale of 0 – 10, with 0 indicating a very poor animal welfare measure)

0 1 2 3 4 5 6 7 8 9 10

Very poor Very good

☐ Don't know

------------------ NY SIDE---------------------------

Sows and gilts and the animal welfare measure 'vacuum chewing and bar biting'

Abnormal behavior in sows and gilts such as vacuum chewing or bar-biting can be associated with hunger and restriction of affective behavior. When assessing this measure it is checked whether vacuum chewing and/or bar biting is present.

27. How would you rate *vacuum chewing and bar biting* as a measure of the underlying state of animal welfare in a sow herd?

(Please rate on a scale of 0 – 10, with 0 indicating a very poor animal welfare measure)

0 1 2 3 4 5 6 7 8 9 10

Very poor Very good

☐ Don't know

----------------- NY SIDE--------------------------

Sows and gilts and the animal welfare measure 'vulva lesions'

Vulva lesions are caused by biting activity of other pigs and show that sows in group housing are not adequately protected whilst feeding. If the animal has either lesions or scars greater than 2 cm or a deformed vulva a welfare problem is registered.

28. How would you rate *vulva lesions* as a measure of the underlying state of animal welfare in a sow herd?

(Please rate on a scale of 0 – 10, with 0 indicating a very poor animal welfare measure)

0 1 2 3 4 5 6 7 8 9 10

Very poor Very good

☐ Don't know

----------------- NY SIDE--------------------------

Finishing pigs and weaners and the animal welfare measure 'lameness'

Lameness is the inability to use one or more limbs in a normal manner. It can vary in severity from reduced ability or inability to bear weight to total recumbency. The pigs are observed from the front, side and back. The video shows a pig suffering from lameness in a severe degree having no ability to bear weight.

Halt gris.avi

29. How would you rate *lameness* as a measure of the underlying state of animal welfare in a flock of finishing pigs or weaners?

(Please rate on a scale of 0 – 10, with 0 indicating a very poor animal welfare measure)

0 1 2 3 4 5 6 7 8 9 10

Very poor Very good

☐ Don't know

------------------ NY SIDE---------------------------

Finishing pigs and weaners and the animal welfare measure 'tail bite'

Tail biting is an abnormal behavior, characterized by one pig's dental manipulation of another pig's tail. If an animal shows signs of wounds, evidence of infection, swelling or bloody appearance, or total or partial loss of the tail including healed lesions, it is registered as a 'tail bite'.

30. How would you rate *tail bite* as a measure of the underlying state of animal welfare in a flock of finishing pigs or weaners?

(Please rate on a scale of 0 – 10, with 0 indicating a very poor animal welfare measure)

0 1 2 3 4 5 6 7 8 9 10

Very poor Very good

☐ Don't know

----------------- NY SIDE--------------------------

Finishing pigs and weaners and the animal welfare measure 'rooting material'

Pigs must be provided rooting material, which must be placed and present in such an amount that more than one pig can use it at the same time. It is required that the material has a high value consisting of either straw, roughage, branches, wood saving, compost, silage, peat or compound materials. Otherwise a welfare problem is registered. This picture illustrates rooting material of a good quality (willow chips).

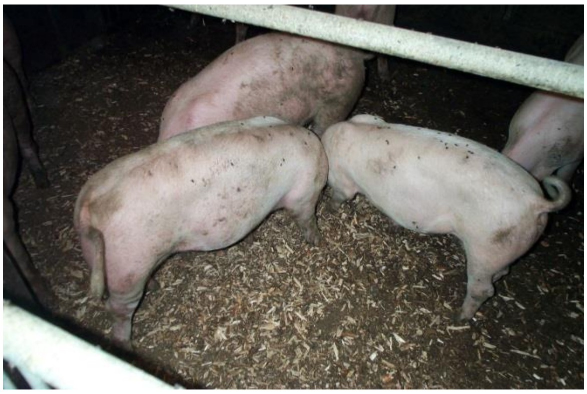

31. How would you rate *rooting material* as a measure of the underlying state of animal welfare in a flock of finishing pigs or weaners?

(Please rate on a scale of 0 – 10, with 0 indicating a very poor animal welfare measure)

0 1 2 3 4 5 6 7 8 9 10

Very poor Very good

☐ Don't know

----------------- NY SIDE--------------------------

In the following questions you will be presented various scenarios of different animal herds suffering from severe to mild welfare problems. The cases vary in levels of severity and proportion of animals being subjected to these conditions.

----------------- NY SIDE--------------------------

Conditions of lameness in herds of Dairy Cattle

The following questions concern various conditions of lameness in herds of dairy cattle. Lameness describes an abnormality of movement and is caused by reduced ability to use one or more limbs in a normal manner. Lameness can vary in severity from reduced ability to inability to bear weight. The term 'lameness in a mild degree' refers to a reduced ability (imperfect temporal rhytm in stride creating a limp) (see video 1).

The term 'severely lame' refers to a strong reluctance to bear weight on one limb, or has more than one limb that is effected (see video 2).

Video 1: Lame in a milder degree

Score 1_lameness.MPG

Video 2: Severely lame

Score 2_lameness.wmv

------------------ NY SIDE-------

Lameness in a herd of Dairy Cattle

**Condition:**<br><br>Lameness in a milder degree: 10 %

1. How would you rate this condition of lameness in a herd of Dairy Cattle?

(Please rate on a scale of 0 – 10, with 0 indicating a completely unacceptable condition)

0 1 2 3 4 5 6 7 8 9 10 ☐ Don't know

Completely unacceptable Highly acceptable

Definition of the severity level

Severe lameness	*Refers to cattle that has a strong reluctance to bear weight on one limb, or has more than one limb that is effected.*
Lameness in a milder degree	*Refers to a cattle that has a reduced ability (imperfect temporal rhytm in stride creating a limp).*

----------------NY SIDE--------------------

Lameness in a herd of Dairy Cattle

Condition:

Lameness in a milder degree: 40 %

2. How would you rate this condition of lameness in a herd of Dairy Cattle?

(Please rate on a scale of 0 – 10, with 0 indicating a completely unacceptable condition)

0 1 2 3 4 5 6 7 8 9 10 ☐ Don't know

Completely unacceptable Highly acceptable

Definition of the severity level

Severe lameness	*Refers to cattle that has a strong reluctance to bear weight on one limb, or has more than one limb that is effected.*
Lameness in a milder degree	*Refers to a cattle that has a reduced ability (imperfect temporal rhytm in stride creating a limp).*

-----------------NY SIDE---------------------

Lameness in a herd of Dairy Cattle

Condition:

Lameness in a milder degree: 70 %

3. How would you rate this condition of lameness in a herd of Dairy Cattle?

(Please rate on a scale of 0 – 10, with 0 indicating a completely unacceptable condition)

0 1 2 3 4 5 6 7 8 9 10 ☐ Don’t know

Completely unacceptable Highly acceptable

Definition of the severity level

Severe lameness	*Refers to cattle that has a strong reluctance to bear weight on one limb, or has more than one limb that is effected.*
Lameness in a milder degree	*Refers to a cattle that has a reduced ability (imperfect temporal rhytm in stride creating a limp).*

-----------------NY SIDE----------------------

Lameness in a herd of Dairy Cattle

Condition:

Severe lameness: 5 %

Lameness in a milder degree: 10 %

4. How would you rate this condition of lameness in a herd of Dairy Cattle?

(Please rate on a scale of 0 – 10, with 0 indicating a completely unacceptable condition)

0 1 2 3 4 5 6 7 8 9 10 ☐ Don't know

Completely unacceptable Highly acceptable

Definition of the severity level

Severe lameness	*Refers to cattle that has a strong reluctance to bear weight on one limb, or has more than one limb that is effected.*
Lameness in a milder degree	*Refers to a cattle that has a reduced ability (imperfect temporal rhytm in stride creating a limp).*

----------------NY SIDE--------------------

Lameness in a herd of Dairy Cattle

**Condition:**<br><br>Severe lameness: 5 %<br><br>Lameness in a milder degree: 40 %

5. How would you rate this condition of lameness in a herd of Dairy Cattle?

(Please rate on a scale of 0 – 10, with 0 indicating a completely unacceptable condition)

0 1 2 3 4 5 6 7 8 9 10 ☐ Don't know

Completely unacceptable Highly acceptable

Definition of the severity level

Severe lameness	*Refers to cattle that has a strong reluctance to bear weight on one limb, or has more than one limb that is effected.*
Lameness in a milder degree	*Refers to a cattle that has a reduced ability (imperfect temporal rhytm in stride creating a limp).*

-----------------NY SIDE----------------------

Lameness in a herd of Dairy Cattle

Condition:

Severe lameness: 5 %

Lameness in a milder degree: 70 %

6. How would you rate this condition of lameness in a herd of Dairy Cattle?

(Please rate on a scale of 0 – 10, with 0 indicating a completely unacceptable condition)

0 1 2 3 4 5 6 7 8 9 10 ☐ Don't know

Completely unacceptable Highly acceptable

Definition of the severity level

Severe lameness	*Refers to cattle that has a strong reluctance to bear weight on one limb, or has more than one limb that is effected.*
Lameness in a milder degree	*Refers to a cattle that has a reduced ability (imperfect temporal rhytm in stride creating a limp).*

-----------------NY SIDE----------------------

Lameness in a herd of Dairy Cattle

Condition:

Severe lameness: 15 %

Lameness in a milder degree: 10 %

7. How would you rate this condition of lameness in a herd of Dairy Cattle?

(Please rate on a scale of 0 – 10, with 0 indicating a completely unacceptable condition)

0 1 2 3 4 5 6 7 8 9 10 ☐ Don't know

Completely unacceptable Highly acceptable

Definition of the severity level

Severe lameness	*Refers to cattle that has a strong reluctance to bear weight on one limb, or has more than one limb that is effected.*
Lameness in a milder degree	*Refers to a cattle that has a reduced ability (imperfect temporal rhytm in stride creating a limp).*

----------------NY SIDE---------------------

Lameness in a herd of Dairy Cattle

**Condition:**<br><br>Severe lameness: 15 %<br><br>Lameness in a milder degree: 40 %

8. How would you rate this condition of lameness in a herd of Dairy Cattle?

(Please rate on a scale of 0 – 10, with 0 indicating a completely unacceptable condition)

0 1 2 3 4 5 6 7 8 9 10 ☐ Don’t know

Completely unacceptable Highly acceptable

Definition of the severity level

Severe lameness	*Refers to cattle that has a strong reluctance to bear weight on one limb, or has more than one limb that is effected.*
Lameness in a milder degree	*Refers to a cattle that has a reduced ability (imperfect temporal rhytm in stride creating a limp).*

-----------------NY SIDE----------------------

Lameness in a herd of Dairy Cattle

Condition:

Severe lameness: 15 %

Lameness in a milder degree: 70 %

9. How would you rate this condition of lameness in a herd of Dairy Cattle?

(Please rate on a scale of 0 – 10, with 0 indicating a completely unacceptable condition)

0 1 2 3 4 5 6 7 8 9 10 ☐ Don't know

Completely unacceptable Highly acceptable

Definition of the severity level

Severe lameness	*Refers to cattle that has a strong reluctance to bear weight on one limb, or has more than one limb that is effected.*
Lameness in a milder degree	*Refers to a cattle that has a reduced ability (imperfect temporal rhytm in stride creating a limp).*

-----------------NY SIDE----------------------

Conditions of diarrhea in a flock of Dairy Calves

The following questions concern various conditions of prolonged infectious diarrhea in 12 month old dairy calves. Bacteria, viruses and/or parasites can cause diarrhea in calves. A mild condition of diarrhea refers to calves suffering from diarrhea, but in a far less severe state, where only few traces of fecal smear are visible.

----------------- NY SIDE--------------------------

Diarrhea in a flock of Dairy Calves

Condition:

Diarrhea in a milder degree: 10 %

10. How would you rate this condition of diarrhea in a flock of dairy calves?

(Please rate on a scale of 0 – 10, with 0 indicating a completely unacceptable condition)

0 1 2 3 4 5 6 7 8 9 10 ☐ Don’t know

Completely unacceptable Highly acceptable

Definition of the severity level

Severe diarrhea	*Refers to a severe prolonged condition of diarrhea*
Diarrhea in a milder degree	*Refers to a milder degree of diarrhea, where only few traces of fecal smear are visible.*

-----------------NY SIDE----------------------

Diarrhea in a flock of Dairy Calves

Condition:

Diarrhea in a milder degree: 45 %

11. How would you rate this condition of diarrhea in a flock of dairy calves?

(Please rate on a scale of 0 – 10, with 0 indicating a completely unacceptable condition)

0 1 2 3 4 5 6 7 8 9 10 ☐ Don't know

Completely unacceptable Highly acceptable

Definition of the severity level

Severe diarrhea	*Refers to a severe prolonged condition of diarrhea*
Diarrhea in a milder degree	*Refers to a milder degree of diarrhea, where only few traces of fecal smear are visible.*

-----------------NY SIDE----------------------

Diarrhea in a flock of Dairy Calves

Condition:

Diarrhea in a milder degree: 80 %

12. How would you rate this condition of diarrhea in a flock of dairy calves?

(Please rate on a scale of 0 – 10, with 0 indicating a completely unacceptable condition)

0 1 2 3 4 5 6 7 8 9 10 ☐ Don't know

Completely unacceptable Highly acceptable

Definition of the severity level

Severe diarrhea	*Refers to a severe prolonged condition of diarrhea*
Diarrhea in a milder degree	*Refers to a milder degree of diarrhea, where only few traces of fecal smear are visible.*

-----------------NY SIDE----------------------

Diarrhea in a flock of Dairy Calves

Condition:

Severe diarrhea: 15 %

Diarrhea in a milder degree: 10 %

13. How would you rate this condition of diarrhea in a flock of dairy calves?

(Please rate on a scale of 0 – 10, with 0 indicating a completely unacceptable condition)

0 1 2 3 4 5 6 7 8 9 10 ☐ Don't know

Completely unacceptable Highly acceptable

Definition of the severity level

Severe diarrhea	*Refers to a severe prolonged condition of diarrhea*
Diarrhea in a milder degree	*Refers to a milder degree of diarrhea, where only few traces of fecal smear are visible.*

-----------------NY SIDE----------------------

Diarrhea in a flock of Dairy Calves

**Condition:**<br>Severe diarrhea: 15 %<br>Diarrhea in a milder degree: 45 %

14. How would you rate this condition of diarrhea in a flock of dairy calves?

(Please rate on a scale of 0 – 10, with 0 indicating a completely unacceptable condition)

0 1 2 3 4 5 6 7 8 9 10 ☐ Don't know

Completely unacceptable Highly acceptable

Definition of the severity level

Severe diarrhea	*Refers to a severe prolonged condition of diarrhea*
Diarrhea in a milder degree	*Refers to a milder degree of diarrhea, where only few traces of fecal smear are visible.*

-----------------NY SIDE----------------------

Diarrhea in a flock of Dairy Calves

Condition:

Severe diarrhea: 15 %

Diarrhea in a milder degree: 80 %

15. How would you rate this condition of diarrhea in a flock of dairy calves?

(Please rate on a scale of 0 – 10, with 0 indicating a completely unacceptable condition)

0 1 2 3 4 5 6 7 8 9 10 ☐ Don’t know

Completely unacceptable Highly acceptable

Definition of the severity level

Severe diarrhea	*Refers to a severe prolonged condition of diarrhea*
Diarrhea in a milder degree	*Refers to a milder degree of diarrhea, where only few traces of fecal smear are visible.*

-----------------NY SIDE----------------------

Diarrhea in a flock of Dairy Calves

Condition:

Severe diarrhea: 30 %

Milder degree: 10 %

16. How would you rate this condition of diarrhea in a flock of dairy calves?

(Please rate on a scale of 0 – 10, with 0 indicating a completely unacceptable condition)

0 1 2 3 4 5 6 7 8 9 10 ☐ Don't know

Completely unacceptable Highly acceptable

Definition of the severity level

Severe diarrhea	*Refers to a severe prolonged condition of diarrhea*
Diarrhea in a milder degree	*Refers to a milder degree of diarrhea, where only few traces of fecal smear are visible.*

-----------------NY SIDE----------------------

Diarrhea in a flock of Dairy Calves

Condition:

Severe diarrhea: 30 %

Milder degree: 45 %

17. How would you rate this condition of diarrhea in a flock of dairy calves?

(Please rate on a scale of 0 – 10, with 0 indicating a completely unacceptable condition)

0 1 2 3 4 5 6 7 8 9 10 ☐ Don't know

Completely unacceptable Highly acceptable

Definition of the severity level

Severe diarrhea	*Refers to a severe prolonged condition of diarrhea*
Diarrhea in a milder degree	*Refers to a milder degree of diarrhea, where only few traces of fecal smear are visible.*

-----------------NY SIDE----------------------

Diarrhea in a flock of Dairy Calves

Condition:

Severe diarrhea: 30 %

Milder degree: 80 %

18. How would you rate this condition of diarrhea in a flock of dairy calves?

(Please rate on a scale of 0 – 10, with 0 indicating a completely unacceptable condition)

0 1 2 3 4 5 6 7 8 9 10 ☐ Don't know

Completely unacceptable Highly acceptable

Definition of the severity level

Severe diarrhea	*Refers to a severe prolonged condition of diarrhea*
Diarrhea in a milder degree	*Refers to a milder degree of diarrhea, where only few traces of fecal smear are visible.*

-----------------NY SIDE----------------------

Conditions of integument alterations in herds of Finishing Pigs and Weaners

The following questions concern various conditions of integument alterations in herds of finishing pigs and weaners. These are defined as abscesses (> 2 cm in diameter), dermatitis (> 2 cm in diameter), wounds (> 2 cm in diameter), swellings (> 2 cm in diameter) and scratches (> 2 cm in length). A severe condition refers to integument alterations above 5 cm or when the animal has more than 10 lesions. A milder condition refers to finishing pigs and weaners having 5-10 lesions or lesions between 2 and 5 cm. The picture below illustrates a pig with scratches and wounds.

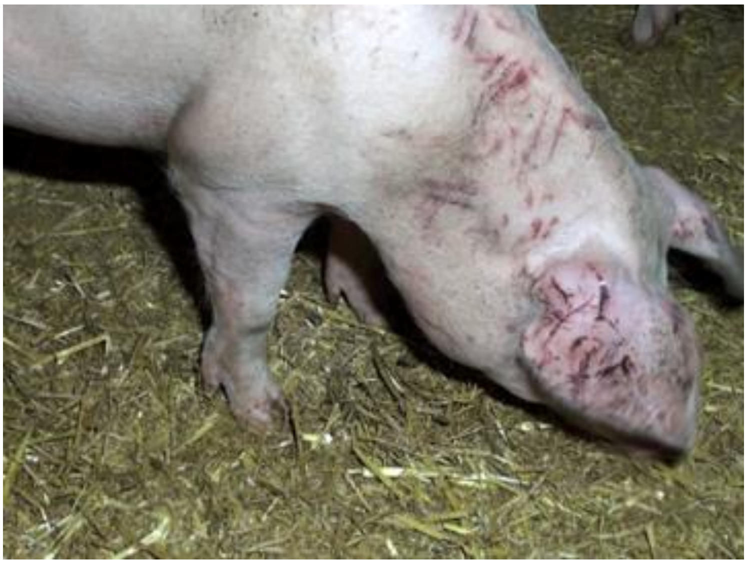

-----------------NY SIDE---------------------

Integument alterations in a herd of Finishing Pigs and Weaners

Condition:

Integument alterations in a milder degree: 40 %

19. How would you rate this condition of integument alterations in a herd of Finishing Pigs and Weaners?

(Please rate on a scale of 0 – 10, with 0 indicating a completely unacceptable condition)

0 1 2 3 4 5 6 7 8 9 10 ☐ Don't know

Completely unacceptable Highly acceptable

Definition of the severity level

Severe integument alterations	*Refers to integument alterations above 5 cm or when the animal has more than 10 lesions.*
Integument alterations in a milder degree	*Refers to integument alterations of 5-10 lesions or lesions between 2 and 5 cm.*

-----------------NY SIDE----------------------

Integument alterations in a herd of Finishing Pigs and Weaners

Condition:

Integument alterations in a milder degree: 75 %

20. How would you rate this condition of integument alterations in a herd of Finishing Pigs and Weaners?

(Please rate on a scale of 0 – 10, with 0 indicating a completely unacceptable condition)

0 1 2 3 4 5 6 7 8 9 10 ☐ Don't know

Completely unacceptable Highly acceptable

Definition of the severity level

Severe integument alterations	*Refers to integument alterations above 5 cm or when the animal has more than 10 lesions.*
Integument alterations in a milder degree	*Refers to integument alterations of 5-10 lesions or lesions between 2 and 5 cm.*

-----------------NY SIDE----------------------

Integument alterations in a herd of Finishing Pigs and Weaners

Condition:

Severe integument alterations: 20 %

21. How would you rate this condition of integument alterations in a herd of Finishing Pigs and Weaners?

(Please rate on a scale of 0 – 10, with 0 indicating a completely unacceptable condition)

0 1 2 3 4 5 6 7 8 9 10 ☐ Don't know

Completely unacceptable Highly acceptable

Definition of the severity level

Severe integument alterations	*Refers to integument alterations above 5 cm or when the animal has more than 10 lesions.*
Integument alterations in a milder degree	*Refers to integument alterations of 5-10 lesions or lesions between 2 and 5 cm.*

-----------------NY SIDE---------------------

Integument alterations in a herd of Finishing Pigs and Weaners

Condition:

Severe integument alterations: 40 %

22. How would you rate this condition of integument alterations in a herd of Finishing Pigs and Weaners?

(Please rate on a scale of 0 – 10, with 0 indicating a completely unacceptable condition)

0 1 2 3 4 5 6 7 8 9 10 ☐ Don't know

Completely unacceptable Highly acceptable

Definition of the severity level

Severe integument alterations	*Refers to integument alterations above 5 cm or when the animal has more than 10 lesions.*
Integument alterations in a milder degree	*Refers to integument alterations of 5-10 lesions or lesions between 2 and 5 cm.*

-----------------NY SIDE----------------------

Integument alterations in a herd of Finishing Pigs and Weaners

Condition:

Severe integument alterations: 20 %

Integument alterations in a milder degree: 40 %

23. How would you rate this condition of integument alterations in a herd of Finishing Pigs and Weaners?

(Please rate on a scale of 0 – 10, with 0 indicating a completely unacceptable condition)

0 1 2 3 4 5 6 7 8 9 10 ☐ Don't know

Completely unacceptable Highly acceptable

Definition of the severity level

Severe integument alterations	*Refers to integument alterations above 5 cm or when the animal has more than 10 lesions.*
Integument alterations in a milder degree	*Refers to integument alterations of 5-10 lesions or lesions between 2 and 5 cm.*

-----------------NY SIDE----------------------

Integument alterations in a herd of Finishing Pigs and Weaners

Condition:

Severe integument alterations: 20 %

Integument alterations in a milder degree: 75 %

24. How would you rate this condition of integument alterations in a herd of Finishing Pigs and Weaners?

(Please rate on a scale of 0 – 10, with 0 indicating a completely unacceptable condition)

0 1 2 3 4 5 6 7 8 9 10 ☐ Don’t know

Completely unacceptable Highly acceptable

Definition of the severity level

Severe integument alterations	*Refers to integument alterations above 5 cm or when the animal has more than 10 lesions.*
Integument alterations in a milder degree	*Refers to integument alterations of 5-10 lesions or lesions between 2 and 5 cm.*

-----------------NY SIDE----------------------

Integument alterations in a herd of Finishing Pigs and Weaners

Condition:

Severe integument alterations: 40 %

Integument alterations in a milder degree: 40 %

25. How would you rate this condition of integument alterations in a herd of Finishing Pigs and Weaners?

(Please rate on a scale of 0 – 10, with 0 indicating a completely unacceptable condition)

0 1 2 3 4 5 6 7 8 9 10 ☐ Don't know

Completely unacceptable Highly acceptable

Definition of the severity level

Severe integument alterations	*Refers to integument alterations above 5 cm or when the animal has more than 10 lesions.*
Integument alterations in a milder degree	*Refers to integument alterations of 5-10 lesions or lesions between 2 and 5 cm.*

-----------------NY SIDE----------------------

Integument alterations in a herd of Finishing Pigs and Weaners

Condition:

Severe integument alterations: 40 %

Integument alterations in a milder degree: 75 %

26. How would you rate this condition of integument alterations in a herd of Finishing Pigs and Weaners?

(Please rate on a scale of 0 – 10, with 0 indicating a completely unacceptable condition)

0 1 2 3 4 5 6 7 8 9 10 ☐ Don't know

Completely unacceptable Highly acceptable

Definition of the severity level

Severe integument alterations	*Refers to integument alterations above 5 cm or when the animal has more than 10 lesions.*
Integument alterations in a milder degree	*Refers to integument alterations of 5-10 lesions or lesions between 2 and 5 cm.*

-----------------NY SIDE---------------------

Body condition score in herds of Sows and Gilts

The next questions concern various levels of body condition score in herds of Sows and Gilts. The body condition provides a clear indication of both the appropriateness of the feed and the effectiveness of the feed delivery system. The severe degree concerning body condition refers to very lean animals, which comprises sows and gilts who visually are very thin, with hips and backbone very prominent. The less severe condition is defined as lean in to a lesser degree, which refers to animals where the the hip bones and backbone are easily felt without any pressure on the palms. In the picture the sow on the left side illustrates a severe body condition score (very lean), while the sow to the right exemplifies a less severe degree of body condition score.

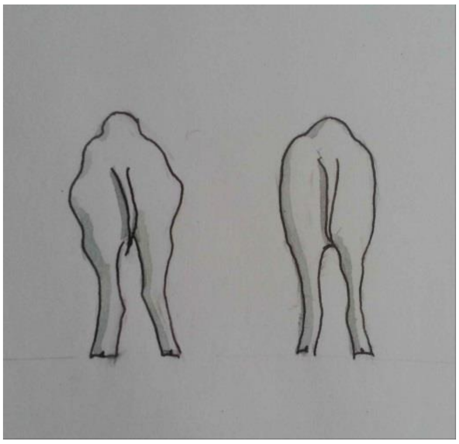

-----------------NY SIDE----------------------

Body condition score in a herd of Sows and Gilts

**Condition:**<br><br>Less severe body condition score: 5 %

27. How would you rate this condition of body condition score in a herd of sows and gilts?

(Please rate on a scale of 0 – 10, with 0 indicating a completely unacceptable condition)

0 1 2 3 4 5 6 7 8 9 10 ☐ Don't know

Completely unacceptable Highly acceptable

Definition of the severity level

Severe body condition score	*Refers to very lean animals who visually are very thin, with hips and backbone very prominent.*
Less severe body condition score	*Refers to lean in a less degree, where the hip bones and backbone are easily felt without any pressure on the palms.*

-----------------NY SIDE----------------------

Body condition score in a herd of Sows and Gilts

Condition:

Less severe body condition score: 10 %

28. How would you rate this condition of body condition score in a herd of sows and gilts?

(Please rate on a scale of 0 – 10, with 0 indicating a completely unacceptable condition)

0 1 2 3 4 5 6 7 8 9 10 ☐ Don’t know

Completely unacceptable Highly acceptable

Definition of the severity level

Severe body condition score	*Refers to very lean animals who visually are very thin, with hips and backbone very prominent.*
Less severe body condition score	*Refers to lean in a less degree, where the hip bones and backbone are easily felt without any pressure on the palms.*

-----------------NY SIDE----------------------

Body condition score in a herd of Sows and Gilts

Condition:

Severe body condition score: 2,5 %

29. How would you rate this condition of body condition score in a herd of sows and gilts?

(Please rate on a scale of 0 – 10, with 0 indicating a completely unacceptable condition)

0 1 2 3 4 5 6 7 8 9 10 ☐ Don't know

Completely unacceptable Highly acceptable

Definition of the severity level

Severe body condition score	*Refers to very lean animals who visually are very thin, with hips and backbone very prominent.*
Less severe body condition score	*Refers to lean in a less degree, where the hip bones and backbone are easily felt without any pressure on the palms.*

-----------------NY SIDE----------------------

Body condition score in a herd of Sows and Gilts

Condition:

Severe body condition score: 5 %

30. How would you rate this condition of body condition score in a herd of sows and gilts?

(Please rate on a scale of 0 – 10, with 0 indicating a completely unacceptable condition)

0 1 2 3 4 5 6 7 8 9 10 ☐ Don't know

Completely unacceptable Highly acceptable

Definition of the severity level

Severe body condition score	*Refers to very lean animals who visually are very thin, with hips and backbone very prominent.*
Less severe body condition score	*Refers to lean in a less degree, where the hip bones and backbone are easily felt without any pressure on the palms.*

-----------------NY SIDE----------------------

Body condition score in a herd of Sows and Gilts

**Condition:**<br><br>Severe body condition score: 2,5 %<br><br>Less severe body condition score: 5 %

31. How would you rate this condition of body condition score in a herd of sows and gilts?

(Please rate on a scale of 0 – 10, with 0 indicating a completely unacceptable condition)

0 1 2 3 4 5 6 7 8 9 10 ☐ Don't know

Completely unacceptable Highly acceptable

Definition of the severity level

Severe body condition score	*Refers to very lean animals who visually are very thin, with hips and backbone very prominent.*
Less severe body condition score	*Refers to lean in a less degree, where the hip bones and backbone are easily felt without any pressure on the palms.*

-----------------NY SIDE----------------------

Body condition score in a herd of Sows and Gilts

**Condition:**<br>Severe body condition score: 2,5 %<br>Less severe body condition score: 10 %

32. How would you rate this condition of body condition score in a herd of sows and gilts?

(Please rate on a scale of 0 – 10, with 0 indicating a completely unacceptable condition)

0 1 2 3 4 5 6 7 8 9 10 ☐ Don't know

Completely unacceptable Highly acceptable

Definition of the severity level

Severe body condition score	*Refers to very lean animals who visually are very thin, with hips and backbone very prominent.*
Less severe body condition score	*Refers to lean in a less degree, where the hip bones and backbone are easily felt without any pressure on the palms.*

-----------------NY SIDE---------------------

Body condition score in a herd of Sows and Gilts

**Condition:**<br>Severe body condition score: 5 %<br>Less severe body condition score: 5 %

33. How would you rate this condition of body condition score in a herd of sows and gilts?

(Please rate on a scale of 0 – 10, with 0 indicating a completely unacceptable condition)

0 1 2 3 4 5 6 7 8 9 10 ☐ Don’t know

Completely unacceptable Highly acceptable

Definition of the severity level

Severe body condition score	*Refers to very lean animals who visually are very thin, with hips and backbone very prominent.*
Less severe body condition score	*Refers to lean in a less degree, where the hip bones and backbone are easily felt without any pressure on the palms.*

-----------------NY SIDE----------------------

Body condition score in a herd of Sows and Gilts

**Condition:**<br>Severe body condition score: 5 %<br>Less severe body condition score: 10 %

34. How would you rate this condition of body condition score in a herd of sows and gilts?

(Please rate on a scale of 0 – 10, with 0 indicating a completely unacceptable condition)

0 1 2 3 4 5 6 7 8 9 10 ☐ Don't know

Completely unacceptable Highly acceptable

Definition of the severity level

Severe body condition score	*Refers to very lean animals who visually are very thin, with hips and backbone very prominent.*
Less severe body condition score	*Refers to lean in a less degree, where the hip bones and backbone are easily felt without any pressure on the palms.*

-----------------NY SIDE----------------------

99. Thanks a lot for taking your time to participate in this survey!

-----------NY SIDE-------------
